# Protein kinases on carbon metabolism: potential targets for alternative chemotherapies against toxoplasmosis

**DOI:** 10.3389/fcimb.2023.1175409

**Published:** 2023-05-23

**Authors:** Denis Amilton dos Santos, Higo Fernando Santos Souza, Ariel M. Silber, Tatiana de Arruda Campos Brasil de Souza, Andréa Rodrigues Ávila

**Affiliations:** ^1^ Laboratório de Pesquisa em Apicomplexa, Instituto Carlos Chagas, Fiocruz, Curitiba, Brazil; ^2^ Laboratory of Biochemistry of Trypanosomes (LabTryp), Departamento de Parasitologia, Instituto de Ciências Biomédicas, Universidade de São Paulo, São Paulo, Brazil; ^3^ Laboratório de Proteômica Estrutural e Computacional, Instituto Carlos Chagas, Fiocruz, Curitiba, Brazil

**Keywords:** kinases, parasite metabolism, drug targets, toxoplasmosis, chemotherapy

## Abstract

The apicomplexan parasite *Toxoplasma gondii* is the causative agent of toxoplasmosis, a global disease that significantly impacts human health. The clinical manifestations are mainly observed in immunocompromised patients, including ocular damage and neuronal alterations leading to psychiatric disorders. The congenital infection leads to miscarriage or severe alterations in the development of newborns. The conventional treatment is limited to the acute phase of illness, without effects in latent parasites; consequently, a cure is not available yet. Furthermore, considerable toxic effects and long-term therapy contribute to high treatment abandonment rates. The investigation of exclusive parasite pathways would provide new drug targets for more effective therapies, eliminating or reducing the side effects of conventional pharmacological approaches. Protein kinases (PKs) have emerged as promising targets for developing specific inhibitors with high selectivity and efficiency against diseases. Studies in *T*. *gondii* have indicated the presence of exclusive PKs without homologs in human cells, which could become important targets for developing new drugs. Knockout of specific kinases linked to energy metabolism have shown to impair the parasite development, reinforcing the essentiality of these enzymes in parasite metabolism. In addition, the specificities found in the PKs that regulate the energy metabolism in this parasite could bring new perspectives for safer and more efficient therapies for treating toxoplasmosis. Therefore, this review provides an overview of the limitations for reaching an efficient treatment and explores the role of PKs in regulating carbon metabolism in *Toxoplasma*, discussing their potential as targets for more applied and efficient pharmacological approaches.

## 1 General aspects of the parasite and the disease

Toxoplasmosis is a worldwide disease with important impacts on human and animal health (Flegr et al., 2014). The etiologic agent of toxoplasmosis is the pathogen *Toxoplasma gondii*, an obligate intracellular parasite with great ability to infect different genera of warm-blooded species, like several species of birds and more than 300 species of mammals, including humans (Hunter and Sibley, 2012). *Toxoplasma gondii* belongs to the phylum Apicomplexa, which includes more than 6,000 species of intracellular parasites associated with several infectious diseases (Morrison, 2008), such as *Plasmodium* (pathogen of malaria), *Cryptosporidium* (pathogens of diarrheal outbreaks), and *Babesia* (pathogen of babesiosis) (Arisue and Hashimoto, 2015).

*Toxoplasma gondii* is an obligate intracellular protozoan that invades new host cells for its survival and proliferation and differentiates into various forms during its life cycle. The tachyzoite is the asexual replicative form that can infect virtually any host cell, while the bradyzoite is the latent form present in tissue cysts (Skariah et al., 2010). The sexual phase of the parasite occurs in the digestive system of felids, the definitive hosts, leading to the formation of the oocysts (with sporozoites) that contaminate soil, water, and food sources (Djurković-Djaković et al., 2019). Intermediate hosts, including humans, can be infected by ingesting food contaminated by oocysts or consuming undercooked meat from infected animals with tissue cysts (Ducrocq et al., 2021).

Immunocompetent individuals are usually asymptomatic; however, severe manifestations of the disease, which can also progress to death, are observed in immunocompromised patients (Robert-Gangneux and Dardé, 2012). The presence of tissue cysts is a survival mechanism of the parasite for evasion of the immune system, prolonging the persistence of the parasite in the host. It is still unknown whether the reactivation of cysts is completely prevented under conditions of immunocompetence or whether the parasite can release immunosuppressive factors, triggering reactivation of the infection (Elsheikha et al., 2020). The reactivation of tissue cysts can lead to serious health problems linked to ocular damage and blindness (Jones and Holland, 2010; Grigg et al., 2015). The occurrence of vertical transmission of parasites during pregnancy can lead to irreversible damage in embryos (Mareze et al., 2019). Studies conducted in France have shown that both prevention and the administration of adequate drugs during pregnancy can reduce newborns’ risk of symptoms and sequelae (Wallon and Peyron, 2015).

Although toxoplasmosis occurs worldwide, seroprevalence rates vary by geographic region. Higher rates are usually verified in people with lower socioeconomic levels or fewer years of schooling (Jones et al., 2014). A study in Germany revealed a global seroprevalence of 55%, and the risk of toxoplasmosis was associated with the consumption of raw meat (Wilking et al., 2016). Low prevalence rates were found in England (17.32%) in pregnant women (Flatt and Shetty, 2013) and a prevalence of 12.3% was found in China in patients with psychiatric disorders (Xiao et al., 2010). In Japan, the presence of anti-*T. gondii* antibodies in pregnant women was 10.3%, and the possibility of infection during pregnancy was only 0.25% (Sakikawa et al., 2012)

The scenario observed for toxoplasmosis in Latin America is unique, with higher infection rates than countries in the Northern Hemisphere. In Mexico, seroprevalence can reach 70% of the population (Caballero-Ortega et al., 2012). In Colombia, a seroprevalence of 29.9% was observed in blood donors (Alberto Betancur et al., 2011) but can reach more than 80% in workers from numerous slaughterhouses (Cortes et al., 2009) or as a result of consuming unboiled water and wild animals (Gómez-Marín et al., 2012). In Cuba, ocular toxoplasmosis can exceed 45% (Bustillo et al., 2015)and in Panama, seroprevalence of 44.41% occurs in pregnant women, representing a high-risk factor for congenital toxoplasmosis (Flores et al., 2021).

The average seroprevalence rate of *T. gondii* in Brazil, the largest country in South America, is above 60%, varying among geographic regions (Pappas et al., 2009). For example, in Fortaleza, a city in the Northeastern region, the general seroprevalence in pregnant women was 68.6% (Sroka et al., 2010) while in the state of Rio Grande do Sul, in the Southern region, a study indicated a positivity of 74.5% (Spalding et al., 2005). In the state of Minas Gerais, in the Southeastern region, the rate increases to 68.3% in rural areas, possibly associated with the consumption of unwashed fruit, contaminated water, undercooked meat, and unpasteurized milk (Antinarelli et al., 2021). The city of Santa Maria, in the Southern region, has observed the largest outbreak of toxoplasmosis in the country, with a high occurrence of congenital infection and severe abnormalities in newborns (Conceição et al., 2021).

Initial studies involving the genetic characterization of *T. gondii* described three clonal lineages, classified as Types I, II, and III (Howe and Sibley, 1995). Type I strains are characterized by a higher virulent profile, causing severe clinical manifestations in humans, such as ocular toxoplasmosis (Khan et al., 2005), psychiatric disorders, and intense inflammatory responses from the host immune system (Halonen and Weiss, 2013). Type II and Type III strains are less virulent and can differentiate into bradyzoites, forming tissue cysts that constitute the latent phase of the infection (Skariah et al., 2010). In South America, cases of ocular toxoplasmosis tend to be more severe, with larger and more numerous lesions than those found in Europe (de-la-Torre et al., 2013). The severe clinical manifestations of toxoplasmosis found in Central and South America can be associated with the highest prevalence of Type I and the occurrence of strains with high genomic diversity and high virulence (Shwab et al., 2018; Hosseini et al., 2019). Type II parasites are widely spread in human populations from North America and Europe, and this type is highly associated with ocular toxoplasmosis in France (Fekkar et al., 2011). A systematic review of studies on clinical samples indicates that the prevalence of *T. gondii* genotypes varies among the continents, with the highest prevalence of Type I strain reported for North and South America and the lowest prevalence for Africa. Type I and Type III were reported more in North and South America (Hosseini et al., 2019)

The genetic diversity of *Toxoplasma* found in South America differs from that found in North America and Europe, where hundreds of atypical genotypes coexist without dominant strains (Shwab et al., 2014). Phylogenetic analyses have indicated that the ancestor of *T. gondii* emerged in the Amazon around 1.5 million years ago. Various factors have contributed to genomic diversification and the emergence of more virulent strains in South America. The great diversity of intermediate hosts, carnivorism between species, and the presence of other felid species (for sexual reproduction) have provided different selective pressures. As a result, the emergence of new lineages increased (Bertranpetit et al., 2017). In addition, the migratory events of the fauna between the Americas allowed the entrance of new genotypes of *Toxoplasma* in North America, and the further passage of animals through the Bering Strait enabled the arrival of the parasite in Asia, Europe, and Africa (Bertranpetit et al., 2017). The current genomic structure of a few strains of *T. gondii* found in the Old World can be attributed to the recent expansion of domestic cats as companion animals and as the primary definitive hosts, where previously only genotypes of parasites more adapted to this animal were more easily dispersed (Müller and Howard, 2016).

*Toxoplasma* is an apicomplexan parasite that has a symbiotic organelle, the apicoplast, essential for its survival due to its vital role in metabolism, mainly in pathways linked to the biosynthesis of fatty acids, isoprenoids, and heme (Van Dooren and Striepen, 2013; Arisue and Hashimoto, 2015). Furthermore, the apicoplast displays specific enzymes with no homologs in the host cells; thus, this organelle could be considered an important potential target for more specific treatments.

## 2 Chemotherapies against *T. gondii*: current drugs and challenges

The pharmacological approaches against toxoplasmosis usually combine two drugs, pyrimethamine and sulfadiazine, which act synergistically on the parasite’s survival and proliferation, inhibiting the folate metabolic pathways (Dunay et al., 2018). However, these drugs are not selective enough against the parasites and affect biochemical pathways in human cells. Adverse effects have been related mainly to pyrimethamine and its inhibition effect on folic acid pathways in tissues with high metabolic activity, such as epithelium and bone marrow. In addition, significant adverse effects can occur independent of the clinical manifestation of the infection (Ben-Harari et al., 2017). A study involving 115 patients with symptoms of encephalitis indicated that 62% suffered side effects due to the use of sulfadiazine, and around a third of the patients abandoned the treatment (Alday and Doggett, 2017). The toxic effects of drugs routinely used are significant, and cases of *Toxoplasma* infections reported in the FDA adverse events reporting system (FAERS) demonstrated that most adverse outcome reports were serious (89%), followed by the adverse outcome of death (11%). The study also confirmed that most adverse outcome reports involved pyrimethamine followed by sulfonamides and that hepatocellular injury, eosinophilia, and systemic symptoms were associated with sulfonamide drug reactions, whereas pancytopenia, neutropenia, and nausea were associated with pyrimethamine (Shammaa et al., 2021).

Sulfadiazine can be substituted for clindamycin in people allergic to sulfa drugs, although the treatment is less effective and has similar adverse effects (Katlama et al., 1996). The combination of sulfamethoxazole and trimethoprim has also shown similar efficacy to pyrimethamine-sulfadiazine. Therefore, it can be used as an alternative in patients who cannot tolerate pyrimethamine or when the drug is unavailable (Rajapakse et al., 2013). However, in addition to side effects, the administration of pyrimethamine and sulfadiazine is associated with rare severe reactions, including agranulocytosis and hepatic necrosis (Alday and Doggett, 2017).

Another significant factor is the need for long-term therapies, lasting from four to six weeks in cases of eye infections and up to one year in patients with congenital infections. Even longer treatments may be necessary since the drugs used are unable to affect bradyzoites and are ineffective in eliminating tissue cysts (Harrell and Carvounis, 2014). Therefore, new therapies should address criteria such as increased selectivity, reduced side effects, elimination of tissue cysts, and reduced drug exposure time.

Studies have proposed that the apicoplast could be an important target for specific drugs since it is considered the parasite’s Achilles Heel due to its vital role in critical metabolic pathways (Kadian et al., 2018). Several compounds have shown efficient inhibition effects on apicoplast pathways. For example, the natural compound thiolactomycin (TLM) is a known selective inhibitor of the Type II fatty acid biosynthesis found in the plant plastids and bacteria, which also occurs in the apicoplast, exhibiting growth inhibition against *Plasmodium falciparum* parasites (Waller et al., 1998). Synthetic analogs of TLM also showed improved antiparasitic effects against *Plasmodium* (Waller et al., 2003), *Trypanosoma cruzi*, and *T. brucei* (Jones et al., 2014).

Another example is the natural compound that disrupts the membrane metalloprotease FtsH1 of apicoplasts, inhibiting organelle biogenesis and leading to apicoplast loss and, consequently, parasite death (Amberg-Johnson et al., 2017). Furthermore, the machinery of apicoplast replication coordinated by a plastid gyrase can be inhibited by ciprofloxacin, leading to profound alterations in the apicoplast and growth inhibition in the parasites (Botté et al., 2012). Finally*, Plasmodium* parasites treated with doxycycline show impaired gene expression, causing defects in apicoplast biogenesis and morphology and leading to the distribution of nonfunctional apicoplasts into daughter merozoites (Dahl et al., 2006), confirming the importance of apicoplast enzymes as potential specific drug targets.

Apicoplasts also play an important role in the parasite’s energy metabolism, containing all subunits of pyruvate dehydrogenase complex (PDH) that is usually found in the mitochondrion in most organisms, catalyzing the conversion of pyruvate to acetyl-CoA, which is then used in Type II fatty acid biosynthesis (Fleige et al., 2007). Since the PDH complex is found in the apicoplast, the mitochondrial conversion of pyruvate to acetyl-CoA is indeed mediated by the branched-chain ketoacid dehydrogenase complex (BCKDH), which has a different substrate in human cells; instead of pyruvate, the human enzyme uses α-ketoacid (Oppenheim et al., 2014). Protein kinases (PKs) regulate both complexes in other eukaryotes, but the kinases regulating PDH and BCKDH in *T. gondii* are still unknown (Ferrarini et al., 2021).

Compounds that inhibit PKs have also been promising alternatives for effectively treating several human pathologies, such as diabetes (Prada and Saad, 2013), cancer (Zhang et al., 2009), and diseases caused by viruses (Buljan et al., 2020) and protozoans (Dichiara et al., 2017). PKs are highly involved in cellular pathways, acting in signal transduction and regulating several biochemical pathways, permitting adaptive cellular responses to environmental changes (Buljan et al., 2020). Drug design strategies have allowed the development of specific kinase inhibitors, with high selectivity against cancer, for example, improving patients’ quality of life and survival (Di Felice et al., 2018).

PKs are interesting targets to be explored against different pathogens such as *Plasmodium*, *Leishmania*, *Trypanosoma*, and *Toxoplasma* because, despite the conservation of PK families among other organisms, there are significant differences in protein identities, in addition to significant structural differences to their closest mammalian homologs (Doerig et al., 2002). Furthermore, drug design strategies are also helpful in constructing specific kinase inhibitors acting as allosteric inhibitors in the PKs regulatory domains, with no interactions against the ATP binding pocket region, which is similar among different kinases (Eglen and Reisine, 2010).

In this way, structural differences found in the pathogen kinases also result in differential affinities to potential kinase inhibitors, opening perspectives to design drugs with appropriate affinity to pathogen kinases, with reduced interactions with host kinases and prevention of unwanted effects (Knockaert et al., 2002).

## 3 Protein kinases: general aspects, classification, and regulatory functions

Rapid responses to internal and external stimuli are fundamental requirements to maintain the life cycle of any organism or cell. Moreover, these responses are required to afford protection to cells with regard to environmental changes, such as determining the correct maintenance of biochemical pathways linked to energy metabolism, storage of nutrients, and communication, constituting a significant survival mechanism (Humphrey et al., 2015).

To provide modulation in biochemical cascades, proteins are subject to reversible post-translational modifications (PTMs) that usually occur through the addition of chemical groups, polypeptides, or direct alterations in amino acid residues, leading to conformational changes in the protein structure, activating or inhibiting catalytic sites, and modulating their biological functions (Wang et al., 2014; Spoel, 2018). Over 200 PTMs are known, including phosphorylation, acetylation, and ubiquitination, rapidly altering protein activity with low metabolic cost (Humphrey et al., 2015). A specialized protein is required for each PTM. Reversible PTMs are possible, which require appropriate enzymes to revert the structural changes and restore the chemical activity of the substrates (Oliveira and Sauer, 2012).

The most studied PTM is phosphorylation, catalyzed by PKs that are key regulators of cellular function; they constitute the largest and most functionally diverse protein family. PKs direct proteins’ activity, location, and function by adding a phosphate moiety to substrate proteins. They also orchestrate the activity of almost all cellular pathways, especially those involved in the transduction and transmission of signals and coordination of complex processes such as the cell cycle (Manning, 2005).

Krebs and Fischer were the first researchers to recognize the biological role of PKs in 1956. These authors isolated a protein able to convert a phosphorylase from the inactive to the active form through a phosphorylation process, elucidating the origin of the phosphate group (using labeled ATP) and determining the optimum pH and the influence of Mn^2+^ in the reaction (KREBS and FISCHER, 1956; KREBS et al., 1959). The discovery of ‘reversible protein phosphorylation’ was a great revolution in biochemistry and led to the authors being awarded the Nobel Prize in Physiology or Medicine in 1992.

PKs phosphorylate a substrate protein’s hydroxyl moiety of serine, threonine, or tyrosine (Cohen, 2002). They constitute a superfamily broadly subdivided into eukaryotic protein kinases (ePKs) and “atypical” protein kinases (aPKs) (Miranda-Saavedra et al., 2012). The ePKs constitute the largest group, subclassified into eight families (AGC, CAMK, CK1, CMGC, RGC, STE, TK, and TKL). This classification highlights the sequence similarity among the catalytic domains and the presence of accessory domains, considering the regulatory characteristics. AGC and CAMK tend to phosphorylate basic residues, while CMGC kinases are usually directed to prolines. TK and TKL phosphorylate tyrosine residues, and STE phosphorylates serine and threonine residues (Parsons et al., 2005). The genes encoding ePKs are highly conserved in their primary amino acid sequences and the three-dimensional structures of their catalytic domains, constituting around 2% of the eukaryotic genome (over 520 proteins) (Manning et al., 2002).

On the other hand, the aPK group is constituted by a small set of protein kinases, subclassified into four families (Alpha, PDHK, PIKK, RIO). These families do not share apparent sequence similarity with ePKs but possess kinase activity (Miranda-Saavedra et al., 2007). The main differences between aPKs are related to low structure similarities, with alterations in regulatory regions and limited interaction binding modes (Kanev et al., 2019), as well as lacking 11 subdomains present in the ePKs (Parsons et al., 2005).

The relationships shown in phylogeny also can be used to predict substrates and biological functions of many uncharacterized kinases (Manning and Cantley, 2002). For example, phylogenetic analyses have revealed that the main family of serine/threonine/tyrosine ePKs is also present in Bacteria and Archaea. The shared characteristics of these PKs in different organisms indicate a monophyletic origin that could lead to the last common ancestor (LUCA). In this evolutionary context, authors have proposed that the appropriate name for this kinase family should be Hanks-type kinases, referring to Hanks’ studies that described this group of kinases in 1988 (Hanks et al., 1988; Stancik et al., 2018). This PK group is also responsible for cellular division, growth, glycolysis, and pathogenicity in *Streptococcus pneumoniae* (Henry et al., 2019), virulence in *Mycobacterium tuberculosis*, and spore germination in *Bacillus subtilis* (Cowley et al., 2004; Shah et al., 2008).

### 3.1 The role of kinases in cell metabolism

PKs coordinate essential cellular pathways. Cellular division is highly regulated by cyclin-dependent kinases (CDKs) and cyclins. Cyclins provide the structural domains that activate the enzymatic activity of CDKs, acting as regulatory subunits (Malumbres, 2014). Quiescent cells (at the G0 phase) can be stimulated through mitotic factors, leading to the synthesis of cyclin D and stimulating CDK4 and CDK6. From there, the retinoblastoma (Rb) protein is phosphorylated, activating cascades that modulate the S phase with DNA synthesis. The end of the S phase is marked by the interaction of cyclin A/CDK2, with phosphorylation of CDK6 and E2F1, driving the cell to the G2 phase. After the G2 phase, the M phase is started by activating CDK1 in association with cyclin B (Otto and Sicinski, 2017; Ding et al., 2020). Several types of kinases are activated in events of DNA damage to promote different responses, including cell cycle arrest, protection of replication forks, control of dNTPs levels, or autophagy (Lanz et al., 2019).

PKs also play a decisive role in cell signal transduction, structuring cellular receptors. Receptor tyrosine kinases (RTKs) are integral membrane proteins represented by 58 proteins in humans. They are structurally disposed of by five regions: extracellular ligand-binding domain, transmembrane and juxtamembrane regions, cytoplasmic domain with tyrosine kinase activity, and a C-terminal tail. The interaction with specific extracellular ligands promotes conformation changes in the receptor, activating the kinase domain and promoting the phosphorylation of downstream proteins, amplifying the external signal and resulting in metabolic changes in the cell (Critchley et al., 2018).

Similarly, MAP kinases were shown to play a significant role in the central nervous system (CNS), connecting neurons and participating in the axonal transport and neuroregeneration process (Asih et al., 2020). Deregulation of MAP kinases can lead to altered gene expression patterns, resulting in serious neurodegenerative damage. For example, the risk of Alzheimer’s and Parkinson’s diseases has increased in patients with alterations in kinase activity. Furthermore, altered PKs in the CNS could generate an inflammatory state leading neurons and glial cells to cell death (Ahmed et al., 2020).

In the context of energy metabolism, the AMP-activated protein kinase (AMPK) controls cellular energy, regulating the dynamic of lipids, glucose, and insulin (Xu et al., 2012). In conditions of fasting, physical exercise, and exposure to cold temperatures, ATP is highly consumed, with increased levels of ADP/AMP in the cell, resulting in AMPK activation. The role of AMPK is directed to reducing cholesterol synthesis, fatty acids, and proteins. This metabolic switch increases the glucose and ATP levels, ensuring the supply of energy compounds for the cell. On the other hand, AMPK is found in an inactive state in cases of obesity in cases of increased levels of nutrients in the organism, which demand strong external stimuli for its activation (Ahmad et al., 2020).

Protein kinase C (PKC) isoforms are involved in lipid metabolism, inflammatory responses, and activation of signaling pathways. In diabetes, defects in PKC contribute to worsening clinical conditions, generating plaque formation in the blood vessel, leading to lumen narrowing and ischemia in the myocardial tissue. However, specific PKC drugs without adverse effects on other kinases have not yet been described (Lien et al., 2021). Therefore, many efforts worldwide have been conducted to discover the phosphorylation sites of essential proteins and their corresponding effector kinases (Bradley et al., 2021).

Drugs that inhibit specific kinases have been developed for treating several diseases, and some are currently in clinical use, such as Imatinib (Gleevec^®^) and Gefitinib (Iressa^®^) for cancer treatment (Mikalsen et al., 2006). Until now, 67 specific kinase inhibitor molecules have already been approved by the FDA for therapeutic purposes (Axtman, 2021). In scientific databases, such as the Protein Data Bank (PDB), protein structures and inhibitory complexes related to more than 300 kinases are available for drug design studies (Laufer et al., 2020). The web computational resource (KinCore) predicts the dynamic states of PKs in association with inhibitors, contributing to the identification of more efficient kinase inhibitors (Modi and Dunbrack, 2022) and opening new avenues for the application of safe chemotherapies against a variety of diseases (Elkoshi, 2021). In addition, Uniprot (www.uniprot.org) provides more than 2,000 three-dimensional structures related to human PKs, which can contribute to developing specific kinase inhibitors for therapeutic applications against apicomplexan parasites.

### 3.2 Protein kinases as potential drug targets in protozoan parasites

There is a broad interest in developing protein kinase inhibitors as potential drugs against various diseases, including kinases as potential targets for new antiparasitic pharmacological strategies (Doerig, 2004). An important number of kinases that play critical and essential roles in the *Toxoplasma* life cycle have been suggested and/or described for Apicomplexa in particular (Gaji et al., 2021).

The CDKs are serine/threonine kinases, and their association with cyclins is the primary mechanism of regulation of CDK activity, in addition to phosphorylation, which also modulates the kinase activity. The genus *Toxoplasma* has more than 20 CDKs. On the other hand, *Plasmodium* and *Cryptosporidium* have less than 10 CDKs each (Rotella, 2012). Interestingly, despite their potential interest as therapeutic targets, only three CDKs have been studied with some detail in *Plasmodium falciparum* (PfPK5, PfPK6, and Pfmrk) (Waters and Geyer, 2003). In addition, all major ePKs groups have been observed, except for receptor guanylate cyclase (RGC) and tyrosine kinases, as well as lacking members of the PDHK (except *T. gondii*) and Alpha aPK families. Furthermore, PKs are conserved among apicomplexan and higher eukaryotes (Miranda-Saavedra et al., 2012). The CAMK group is a family of serine/threonine kinases that mediate many of the second messenger effects of Ca^2+^ and is the second-largest kinase group in apicomplexan (Talevich et al., 2012) Regarding the group of calcium-dependent protein kinases (CDPKs) that participate in invading new host cells in *T. gondii* (Kieschnick et al., 2001) are highly related to other proteins found in plants and algae. However, there are no similar proteins in mammals, opening the perspective for the design of specific drugs, like bumped kinase inhibitors (BKIs), which interfere with the activity of CDPK1, blocking the parasite invasion process *in vitro* (Ojo et al., 2010). Similarly, the inhibitor H89, an ATP-competitive compound, reduces the kinase activity of the cAMP-dependent protein kinase (PKA) and, consequently, the replication of tachyzoites in the parasitophorous vacuole (Kurokawa et al., 2011). Finally, other *Toxoplasma* kinases may also be critical pharmacological targets, such as the kinases that participate in immune manipulation (ROP16, 18, and 38), the cell cycle (TgNEK1, TgCK1, TgTPK2), the conversion of tachyzoites to bradyzoites, and those that control the metabolic responses to stress (TgMAPK1, TgPKA-C, and TgEIF2K) (Kato et al., 2012).

In the context of more effective drugs against *Toxoplasma* parasites, some biochemical characteristics involving PKs that participate in the carbon metabolism are only found in this parasite and, for this reason, could become attractive targets for new therapies. Although the TCA cycle is essential for intracellular and extracellular tachyzoites (MacRae et al., 2012), changes in ATP homeostasis occur in extracellular parasites, leading to high dependence on the glycolytic pathway rather than on the oxidative phosphorylation of mitochondria (Shukla et al., 2018). Similarly, erythrocytic stages of *Plasmodium* are also highly dependent on aerobic glycolysis as the major route to ATP production (Salcedo-Sora et al., 2014). Furthermore, in cases where glutamine is the major carbon source, it is catabolized *via* the TCA cycle and used to produce glycolytic intermediates (Lyu et al., 2023). Glycolysis is also essential for parasite egress (Huynh and Carruthers, 2022). In general, glycolysis is the primary source of ATP in the parasites, despite the fact that the mitochondrial pathway is essential for tachyzoites. This dependence on glycolysis is also found in cancer cells that repress the mitochondrial TCA cycle by overexpression of dehydrogenase kinases (PDKs), blocking the activity of pyruvate dehydrogenase complex (PDH) (Yu et al., 2017). DCA is a potent PDK inhibitor in tumor cells that revert the glycolytic phenotype and contribute to cell death in its cells (Michelakis et al., 2008). DCA treatment in *Toxoplasma* causes inhibitory effects in tachyzoites and affects the mitochondrial activity in these parasites, which might indicate that PDK could be the DCA target, regulating the TCA cycle (Ferrarini et al., 2021). However, the PDH complex is present in the apicoplast, distinct from the mitochondrial localization observed in most eukaryotes. In *Toxoplasma*, the absence of the PDH complex in mitochondria was compensated by the BCKDH complex, which took over the functions initially carried out by the canonical PDH (Oppenheim et al., 2014). Two putative mitochondrial kinases (PDK/BCKDK) are reported in the *Toxoplasma* parasites, which are located in the mitochondria. It is still unknown whether these kinases could regulate the BCKDH complex (Ferrarini et al., 2021), and there is still no evidence of potential kinases regulating the PDH complex in the apicoplast. Thus, further insights into the specific features of those PKs that can be useful for new drug strategies are still needed to understand the role of kinases in regulating carbon metabolism.

Nevertheless, it is important to consider that most of the data have been described on tachyzoites; on the other hand, knowledge about carbon metabolism in bradyzoites is still limited, mainly due to experimental constraints. Different studies indicate that glycolytic degradation of glucose also plays an important role in bradyzoites, in which they depend on the turnover of storing polysaccharide amylopectin by glycogen phosphorylase (Coppin et al., 2003), with its formation induced by a few electron transport inhibitors (Tomavo and Boothroyd, 1995; Ferreira Da Silva et al., 2008). Recently, the development of an *in vitro* system developing bradyzoites enabled measurements of bradyzoite metabolites *via* mass spectrometry, revealing lower levels of several TCA cycle-associated metabolites that are consistent with a lower reliance on this pathway (Christiansen et al., 2022). These scalable *in vitro* systems can be useful in providing new data regarding bradyzoite metabolism and identifying PKs and other enzymes specific to glycolytic metabolism in bradyzoites, improving the selectivity of chemotherapy strategies and reducing the side effects. If future studies demonstrate that the overexpression of PDK in bradyzoites is responsible for the low activity of the TCA cycle in this stage, perhaps the DCA treatment would revert the reduced activity of mitochondria and generate a lethal effect due to metabolic disequilibrium, like reversion of the Warburg effect in cancer cells (Ferrarini et al., 2021). Although DCA was not tested on bradyzoites, it is not toxic to human cells; therefore, further studies could open perspectives of repositioning kinase inhibitors used in other diseases for toxoplasmosis therapy.

### 3.3 Regulatory kinases in the carbon metabolism of apicomplexan parasites

PKs that coordinate glycolysis in the parasites could be important targets to screen for new treatments due to the differences found with regard to mammalian cells. In addition, glycolysis is an essential pathway for the parasite life cycle, and disruption of this pathway disturbs tachyzoite replication and the formation of tissue cysts, constituting an essential target for treatments (Shukla et al., 2018).

**Figure 1:**
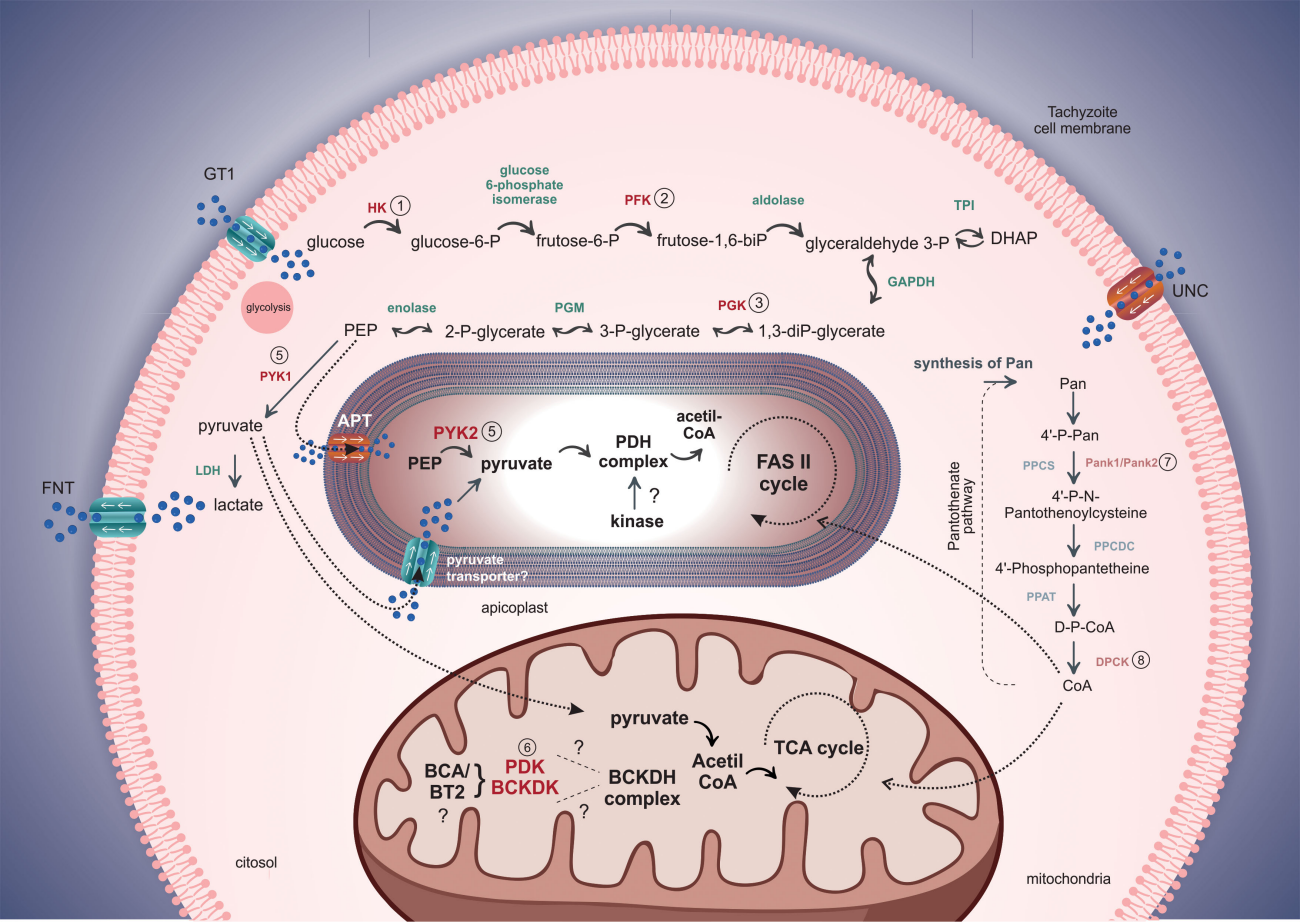
Schematic model of the role of protein kinases in glycolysis, the TCA cycle, and the pantothenate pathways in *T. gondii*. The GT1 transporter is responsible for the internalization of glucose to the cytosol. Kinases in the cytosol, apicoplast, and mitochondria are highlighted and listed. The apicoplast displays a PEP transporter represented as APT in *Toxoplasma* and TPT in *Plasmodium*. The extrusion of excessive lactate and pyruvate produced by glycolysis is exported by the FNT transporter, contributing to the acidification of the parasitophorous vacuole and parasite release. Tachyzoites can synthesize or import pantothenate (Pan) from the host cell, but the Pan transporter is still uncharacterized (UNC). Besides the kinases that act in glycolysis, mitochondria, and apicoplast, kinases in the pantothenate pathway are also important in converting pantothenate to coenzyme-A (CoA) used in other biochemical pathways.

After the invasion, the replicative niche of *Toxoplasma* is constituted by the parasitophorous vacuole (PV), a specialized organelle responsible for tachyzoite protection, manipulating signaling pathways, and acquiring soluble nutrients from the host cell as amino acids, sugars, and nucleotides (Martin et al., 2007). **Figure 1** depicts the kinases regulating different metabolic pathways.

#### 3.3.1 Glycolysis in cytosol

Glucose is internalized by the hexose transporter GT1 distributed on the parasite surface and is not restricted to a particular site in the cell membrane (Pomel et al., 2008). The first stage of glycolysis is mediated by hexokinase (1-HK), an enzyme responsible for converting glucose to glucose-6-phosphate.

Sequence analysis has revealed considerable similarity between *T. gondii* HK and *Plasmodium falciparum* HK but low similarity (> 35%) between parasite and host HKs (Saito et al., 2002). In *Plasmodium vivax*, HK exhibits a homotetrameric structure, unlike that found in human HKs that are organized in monomers or dimers. The organization of the tetrameric structure in *Plasmodium* is related to the most extensive hydrophobic interactions in peripheral regions, generating solid interactions in the interfaces of the kinase. In contrast, these regions are replaced by polar residues (Srivastava et al., 2020). *Toxoplasma* knockout parasites for HK cannot catalyze glucose phosphorylation, even under supplemented glucose conditions, displaying defects in the formation of tissue cysts in the nervous system of mice and alterations in the invasion process of new host cells (Shukla et al., 2018).

*Cryptosporidium* (Apicomplexa) also shows a unique and highly divergent HK to humans and *Toxoplasma*, with the distinctive capacity to use other NTPs besides ATP, unlike other organisms whose HKs are ATP-dependents (Yu et al., 2014).

After phosphorylation mediated by HK, the glucose-6-phosphate is converted to fructose-6-phosphate by the glucose-6-phosphate isomerase. From this point in glycolysis, the differences between the enzymes from parasites and mammalian cells become more marked. The remaining enzymes of glycolysis (from phosphofructokinase to pyruvate kinase) are expressed in two isoforms in the parasites, including isoforms present in the apicoplast (Fleige et al., 2007).

The third stage of glycolysis is mediated by phosphofructokinase (2- PFK), which uses fructose-6-phosphate as a substrate to generate fructose-1,6-biphosphate. Two PFKs are reported in *T. gondii:* PFK1, dependent on ATP, and PFK2, dependent on pyrophosphate as a phosphate donor. While PFK1 is not essential for parasite proliferation, the conditional knockout of PFK2 leads to growth arrest and an increase of pyrophosphate levels in the cytosol, which is in high amounts in the cell, as well as reduces protein synthesis in the tachyzoites (Yang et al., 2022).

*Toxoplasma* PFK2 differs from the ATP-dependent PFK found in the host cells because it differs in subunit structure and catalytic kinetics, constituting a potential drug target. Analogs of phosphonic acid showed inhibitory effects in the kinetic activity of PFK2, inhibiting parasite growth and protective effects in treated host cells (Peng et al., 1995). Mutations in *Plasmodium* PFKs confer drug resistance to antimalarial drugs because they redirect the glycolytic metabolites to the pentose phosphate pathway and improve the parasite’s survival (Fisher et al., 2020). Inhibitory compounds directed to PFKs provide strong effects in other parasites, such as trypanosomes, blocking the glycolytic pathways and leading to parasite death and the complete cure of infected mice, with no side effects to the host (McNae et al., 2021).

The enzyme aldolase mediates the conversion of fructose-1,6-biphosphate to glyceraldehyde 3-phosphate, and the transformation of glyceraldehyde-3-phosphate to 1,3-diphosphoglycerate is mediated by the glyceraldehyde 3-phosphate dehydrogenase (GAPDH), although they are not regulated by kinases (Fleige et al., 2007). It has also been proposed that glyceraldehyde 3-phosphate could be converted to dihydroxyacetone-phosphate (DHAP) in a reversible reaction, with DHAP imported to the apicoplast for metabolization in the isoprenoid pathway (Nair et al., 2011).

Aldolase-deficient *Toxoplasma* strains show growth inhibition due to the accumulation of toxic intermediates from upstream stages of glycolysis, with no interference in the motility and invasion mechanisms (Shen and Sibley, 2014). GAPDH is also essential to parasite metabolism, with parasite growth impairment when the protein is absent through knockout genetic strategies (Dubey et al., 2017). It has been found that *Toxoplasma* enzymes triose phosphate isomerase II (TPI-II), glyceraldehyde-3-phosphate dehydrogenase II (GAPDH-II), and phosphoglycerate kinase II (PGK II) are in the apicoplast and play an important role in the isoprenoids and fatty acids biosynthesis (Niu et al., 2022).

Phosphoglycerate kinase (3-PGK) is the third kinase found in glycolysis, present in two isoforms in Toxoplasma to catalyze the conversion from 1,3-biphosphoglycerate to 3-phosphoglycerate (3-PGA) (Smith et al., 2011). In Plasmodium falciparum, PGK showed a 61% similarity to human PGK, with conserved regions implicated in interaction with the substrate, cofactors, and catalysis. However, it offers higher affinity interaction with ATP and 3-PGA (1.5- to 2.0-fold more, respectively) and lower thermal stability than the human homolog (Hicks et al., 1991; Pal et al., 2004).

Structural differences in PGKs from humans and parasites are related to the acetylation profile of these kinases. In humans, acetylation of PGK occurs in 13 lysines (Choudary et al., 2009), while five lysines are acetylated in intracellular parasites, and only one is found in extracellular parasites (Xue et al., 2013), indicating a possible regulatory function. Although there are no reports about PGK inhibitors for Toxoplasma, the compound adenylyl 1,1,5,5-tetrafluoromethane-1,5-bisphosphonate efficiently occupied the bind sites of the *Trypanosoma* PGK, inhibiting the kinase through conformational changes (Bernstein et al., 1998). PGK is also considered a potential target for cancer therapy, where the expression levels could indicate tumor progression and the existence of a multidrug resistance profile in the tumor cells (Liu et al., 2022).

The two subsequent stages of glycolysis are conducted by phosphoglycerate mutase (PGM), catalyzing the interconversion of 3-phosphoglycerate to 2-phosphoglycerate, with enolase using the 2-phosphoglycerate as a substrate to form phosphoenolpyruvate (PEP) (Hargrave et al., 2019). Studies using the mice model have indicated that recombinant PGM2 from Toxoplasma can induce protective effects in the animals, with improved survival outcomes and a reduction in the number of tachyzoites found in brain and liver tissues (Wang et al., 2016). Toxoplasma shows two isoforms of enolase (enolase 1, ENO1, and enolase 2, ENO2). ENO1 is only expressed in bradyzoites and shows only one-third of the catalytic activity of ENO2. In addition to the activity in glycolysis, ENO1 and ENO2 also exhibit a regulatory function linked to gene expression and are also found in the parasite nucleus (Dzierszinski et al., 2001; Ruan et al., 2015).

#### 3.3.2 FAS II cycle in apicoplast

PEP, generated by the enolase enzyme, can be directed along two distinct routes, following the glycolytic pathway in the cytoplasm, or imported to the apicoplast through plant-like transporters, APT in Toxoplasma or TPT in Plasmodium (Shunmugam et al., 2022). *Toxoplasma gondii* exhibits two isoforms of pyruvate kinases (4-PYK1 and 5-PYK2) responsible for converting PEP to pyruvate in cytosol and apicoplast, respectively (Xia et al., 2019). It has been proposed that pyruvate produced by PYK1 in the cytosol could also be imported to the apicoplast, although its transporter still needs to be confirmed and characterized (Fleige et al., 2007; Xia et al., 2019). PYK1 plays a greater role than PYK2 in parasite metabolism. Depletion of PYK2 does not significantly affect the parasite, but disruption of PYK1 leads to the accumulation of upstream metabolites from glycolysis, reducing ATP production. Disruption of both kinases (PYK1 and PYK2) generates complete growth arrest due to the loss of the apicoplast, an organelle essential to the parasite’s survival (Xia et al., 2019).

Other steps in pyruvate parasite metabolism present significant differences related to mammalian organisms. Pyruvate dehydrogenase complex (PDH), usually found in the mitochondria of other organisms, mediates the metabolization of pyruvate and acetyl-CoA production in the apicoplast, which is then used in Type II fatty acid biosynthesis (FAS II pathway). In the absence of the mitochondrial PDH complex, the BCKDH complex assumes the canonical function of PDH, promoting the conversion of pyruvate to acetyl-CoA, which is then metabolized through the TCA cycle (Oppenheim et al., 2014). It is still unknown if the apicoplast PDH could be regulated by kinases as usually observed in other organisms (Wang et al., 2021), which could also constitute an essential target for use in antiparasitic drugs.

#### 3.3.3 The TCA cycle in mitochondria

In mammalian cells and organisms, the pyruvate produced by pyruvate kinase in the last stage of glycolysis is directed to the mitochondria. In this organelle, pyruvate is decarboxylated by PDH in an irreversible reaction, resulting in the production of acetyl-CoA, CO_2_ release, and the reduction of NAD^+^ to NADH. The acetyl-CoA is then metabolized through the tricarboxylic acid (TCA) cycle in the mitochondria (Anwar et al., 2021). The PDH complex is present in eukaryotes and prokaryotes and is structured through multiple copies of three catalytic subunits: pyruvate dehydrogenase (E1), dihydrolipoamide acetyltransferase (E2), and dihydrolipoamide dehydrogenase (E3). Higher eukaryotes also have the dihydrolipoamide dehydrogenase binding protein (E3BP) subunit. This dehydrogenase complex is controlled by regulatory enzymes such as pyruvate dehydrogenase kinases (with four isoforms reported in humans) and pyruvate dehydrogenase phosphatases (PDP, with two isoforms in humans) (Patel et al., 2014). In non-tumoral mammalian cells, the TCA cycle and oxidative phosphorylation (OXPHOS) in the mitochondria produce significant amounts of ATP from pyruvate. However, even in normal oxygen conditions, tumor cells use glycolysis as the main route for making ATP, inhibiting the mitochondrial TCA cycle and, consequently, the OXPHOS, avoiding oxidative damage and apoptosis and ensuring survival (Anwar et al., 2021). In tumor cells, the Warburg effect is observed, corresponding to a switch from glycolysis controlled by the Pasteur effect to aerobic glycolysis triggered by the pyruvate’s overexpression of dehydrogenase kinases (PDKs). These PDKs promote the phosphorylation of mitochondrial PDH, blocking the formation of acetyl-CoA from pyruvate, its entry into the TCA cycle, and, therefore, the OXPHOS. Such accumulation of carbon compounds permits cell growth and metastasis (Stacpoole, 2017).

Remarkably, reversing the Warburg effect in cancer metabolism is possible using the small molecule inhibitor dichloroacetate (DCA). The mechanism of action of the drug is focused precisely on the inhibition of PDKs, restoring the activity of the PDH complex and mitochondrial functions, and consequently, increasing intracellular ROS levels, the susceptibility to other drugs, as well as stopping the progression of tumor growth (Tataranni and Piccoli, 2019). DCA can be administered orally, and due to its inhibitory effect on PDKs, increasing the pyruvate flux in mitochondria, promoting the TCA cycle, and indirectly the OXPHOS results in the inhibition of tumor growth both *in vitro* and *in vivo* (Michelakis et al., 2008). Therefore, disruption of the PDK/PDH axis is the key to the tumor elimination mechanism of DCA, inducing apoptosis in tumor cells without affecting non-tumor cells. Thus, PDK became a therapeutic target in oncology (Sutendra and Michelakis, 2013).

Metabolism in apicomplexan parasites is also highly dependent on the glycolysis pathway, suppressing mitochondrial metabolism and accumulating intermediate metabolites produced by glycolysis that support rapid proliferation (Salcedo-Sora et al., 2014). Due to TCA cycle repression, the pyruvate accumulated in the cytosol is then converted to lactate by the enzymatic activity of two lactate dehydrogenases (LDH1 and LDH2) and used as an energy source by fermentative processes in the parasite (Xia et al., 2018). The exportation of lactate by the FNT transporters has an important role in the acidification of the parasitophorous vacuole, contributing to the natural egress of the parasites (Huynh and Carruthers, 2022). Inhibitory drugs for the FNT transporters reduce the exportation of lactate, leading to rapid cytosol acidification and cell death in the parasites (Walloch et al., 2021).

The reduction of the TCA cycle observed in the parasites also could be controlled by the PKs. Cluster analyses have revealed the presence of two putative kinases (6- PDK/BCKDK) that may be regulating the mitochondrial carbon metabolism in *T. gondii*, which DCA could target. Treating infected cells with DCA also produced the intracellular parasite’s death without noticeable biological effects on the host cells. It is important to note that it has not been formally demonstrated that the DCA target in the parasites is indeed a kinase (Ferrarini et al., 2021).

In addition to DCA, the compound known as BT2 (3,6-dichlorobenzo[b]thiophene-2-carboxylic acid) is a kinase inhibitor directed to another kinase (BCKDK) in other models. It could target the BCKDK in the parasite mitochondria, indirectly promoting the activity of the BCKDH. Thus, the BCKDK also could be an exciting target for drugs against apicomplexans. BT2 is a potent allosteric inhibitor of BCKDK. Although BCKDK and PDKs share similar structures, the ligand-binding allosteric pocket in BCKDK (412Å^3^ volume) is two to four times larger than their counterparts in PDKs (90Å^3^–213Å^3^) (Zhou et al., 2019). BT2 belongs to a family of benzothiophene derivatives, several of which have already been reported as enzyme inhibitors. It has been identified as a novel BCKDK inhibitor for therapeutic approaches. It reduces the BCAA/BCKA concentrations in maple syrup urine disease (MSUD) and is used to treat obesity and type 2 diabetes (Tso et al., 2014). The treatment of the C2C12 myoblast with BT2 blocks BCKDK, causing the activation of branched-chain amino acids catabolism and consequently suppressing cell proliferation and differentiation (Sato et al., 2020). Inhibition of BCKDK was sufficient to enhance BCKDH activity across tissue types in insulin-resistant, diet-induced obese mice (Zhou et al., 2019). Additionally, benzothiophene carboxamide derivatives are potent slow-binding inhibitors of *Plasmodium* enoyl-acyl carrier protein (ACP) reductase (PfENR), thus suggesting their use as an important potential antimalarial drug (Banerjee et al., 2011) Moreover, benzothiophene derivatives have also shown inhibitory effects in the proliferation of *T. gondii* (Rosada et al., 2019).

#### 3.3.4 Pan synthesis in the cytosol

Beyond the pathways of glycolysis involving the apicoplast and mitochondria, the pantothenate pathway is another important target for applied therapies against the parasites. Pantothenate (Pan, vitamin B5) is an essential precursor for the synthesis of Coenzyme A (CoA), which has a role in gene regulation, the TCA cycle, heme, and fatty acid biosynthesis (Lunghi et al., 2022). While animals acquire Pan only through diet, the apicomplexan parasites have the machinery to produce Pan, becoming essential for the pathogen parasite and a particular target against chronic infection (de Vries et al., 2021). *Toxoplasma* parasites show two isoforms of Pan kinases (7- Pank1 and Pank2) that promote the phosphorylation of Pan to 4’-phosphopantothenate (4’-P-P-Pan). In this pathogen, Pank1 and Pank2 act in a heterodimer complex, unlike in humans, where Pank enzymes exist only as a homodimer and are necessary for tachyzoite proliferation (Tjhin et al., 2021). The last step of CoA biosynthesis occurs through the conversion of phospho-CoA (D-P-CoA) to CoA and is also catalyzed by dephospho-coenzyme A kinase (8- DPCK) (Fletcher et al., 2016). DPCK is in the cytosol of *Toxoplasma* and in the apicoplast of *Plasmodium* (de Vries et al., 2021). *Plasmodium* parasites subjected to apicoplast disruption (mediated by drug strategies) continue to express DPCK, with biochemical activity inside the vesicles derived from the apicoplast rupture. The DPCK remains active inside these vesicles and supplies the parasites with essential metabolites, contributing to parasite survival, also indicating a critical target that could be explored in new drug studies looking for kinase inhibitory strategies (Swift et al., 2021).

Then, the distinctive characteristics found in different PKs in the apicomplexans could open critical perspectives to applied chemotherapies focused on the complete characteristics that differentiate them from host kinases.

## 4 Conclusion

**Figure 2:**
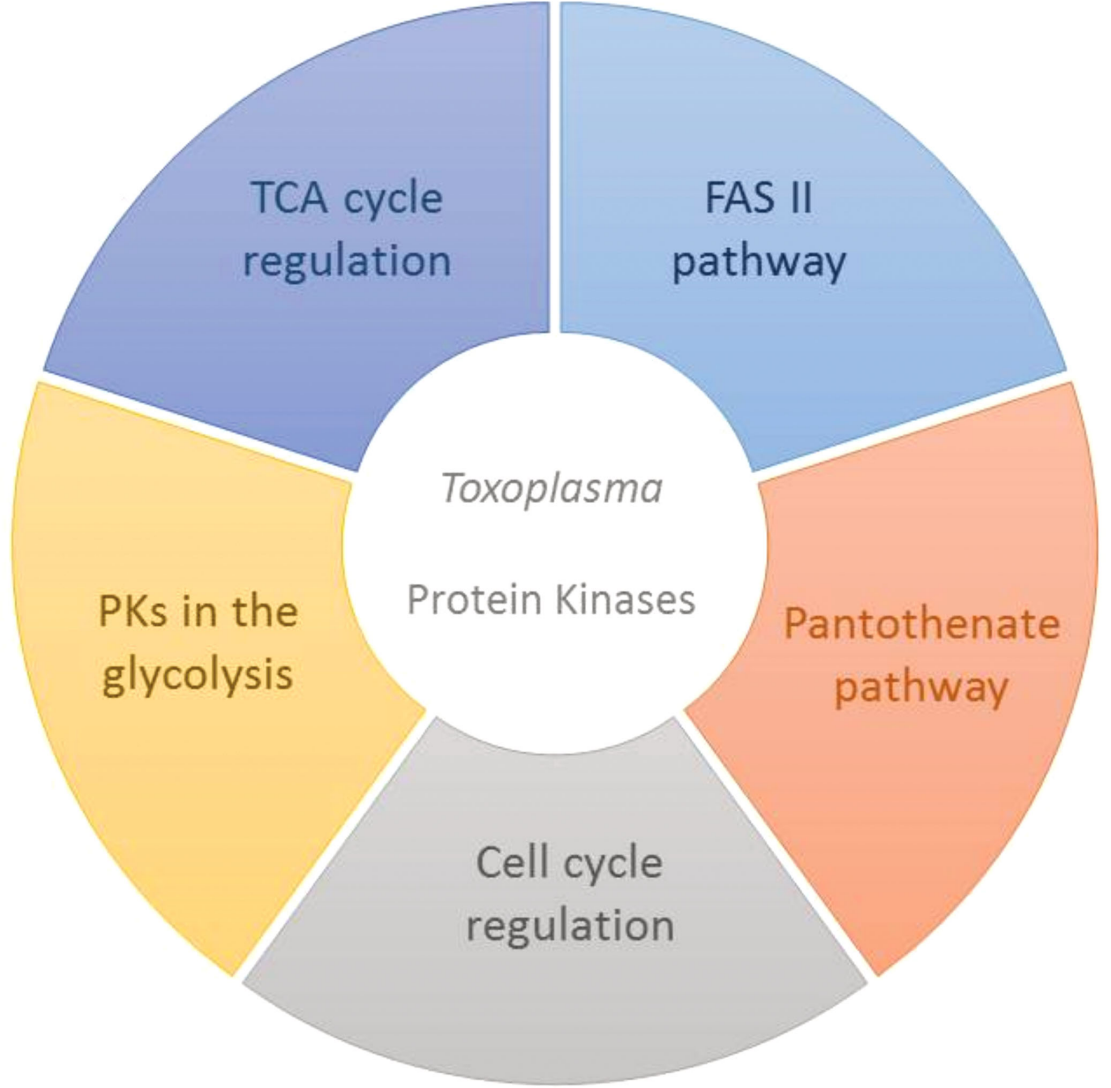
Important protein kinases as possible targets for more effective therapies in *Toxoplasma gondii*. Different pathways are controlled by kinases in the metabolism of the parasites, and the distinctive characteristics found in these proteins may bring significant useful information for new strategies, such as highly selective kinase inhibitors, especially in relation to toxoplasmosis, which still has no definitive cure.

PKs regulate essential aspects of cell metabolism. Therefore, they have become potential targets for effective therapies in different human diseases caused by protozoan pathogens, mainly when they are structurally different from human cells or involved in the distinctive metabolic pathways of those microorganisms. Different routes could be explored for new kinase inhibitors (**Figure 2**) as the CDKs coordinate the parasite cycle. Still, the specificities found in the PKs regulating the parasites’ energy metabolism could offer new perspectives for safer and more effective therapies against *T*. *gondii* parasites.

The research on structural differences, specific kinetic activities, and binding sites found in the kinases PFK, PGK, and PYK in parasite glycolysis can shed light on more detailed and non-toxic chemotherapies. For example, inhibiting PFK2 restricts the formation of metabolites in an early stage of glycolysis and increases pyrophosphate levels to achieve toxic effects on the parasite. Various acetylation sites in parasite PGK could serve as potential sites for specific compounds since acetylated residues are distinct from the host PGK in both proliferative and latent parasites. The combination of strategies aiming to block both PYK1 and PYK2 in the parasites also leads to the suppression of glycolysis and apicoplast loss in the parasites, suppressing the essential FAS II pathway. Furthermore, the presence of isoforms with a possible location in the apicoplast may also indicate the presence of still unknown metabolic routes or catalytic activities, which need to be better evaluated.

The regulatory kinase for PDH in the apicoplast and whether the mitochondrial BCKDH is regulated by the kinases PDK and/or BCKDK remain unclear. Then, elucidating the regulatory kinases of those pathways would help to understand the mechanism of DCA inhibitory effects already reported for tachyzoites.

Other potential important targets in the parasites are the kinases in the pantothenate pathway, which are responsible for producing coenzyme-A (CoA) as the end product. Inhibition of its kinases would indirectly affect the activities of PDH (in the apicoplast) and BCKDH (in mitochondria), since these complexes use CoA in the conversion of pyruvate to acetyl-CoA, affecting both the TCA cycle and FAS II biosynthesis, as well as the role of CoA in other cellular regulatory functions.

Exploring the specificities that differentiate the parasite PKs from the host enzymes will bring information to help develop compounds with high affinity to parasite proteins, trying to reduce the side effects of traditional chemotherapies, and find compounds that would eliminate the latent forms that serve as a reinfection source in patients.

## Author contributions

DS: conceptualization and writing – original draft, writing — review, and editing. HS: writing – original draft, writing — review and editing. AS: writing – original draft, writing — review and editing. TS: writing – original draft, writing — review and editing. AÁ: conceptualization, writing — original draft, writing — review and editing. All authors contributed to the article and approved the submitted version.
